# The Distinctive Evolution of *orfX Clostridium parabotulinum* Strains and Their Botulinum Neurotoxin Type A and F Gene Clusters Is Influenced by Environmental Factors and Gene Interactions via Mobile Genetic Elements

**DOI:** 10.3389/fmicb.2021.566908

**Published:** 2021-02-26

**Authors:** Theresa J. Smith, Charles H. D. Williamson, Karen K. Hill, Shannon L. Johnson, Gary Xie, Fabrizio Anniballi, Bruna Auricchio, Rafael A. Fernández, Patricia A. Caballero, Paul Keim, Jason W. Sahl

**Affiliations:** ^1^Pathogen and Microbiome Institute, Northern Arizona University, Flagstaff, AZ, United States; ^2^Los Alamos National Laboratory, Los Alamos, NM, United States; ^3^Department of Food Safety, Nutrition and Veterinary Public Health, National Reference Centre for Botulism, Istituto Superiore di Sanità, Rome, Italy; ^4^Área Microbiología, Departamento de Patología, Universidad Nacional de Cuyo, Mendoza, Argentina

**Keywords:** *Clostridium parabotulinum*, neurotoxin, plasmids, *orfX*, *lycA*, *arsC*, *pulE*

## Abstract

Of the seven currently known botulinum neurotoxin-producing species of *Clostridium*, *C. parabotulinum, or C. botulinum* Group I, is the species associated with the majority of human botulism cases worldwide. Phylogenetic analysis of these bacteria reveals a diverse species with multiple genomic clades. The neurotoxins they produce are also diverse, with over 20 subtypes currently represented. The existence of different *bont* genes within very similar genomes and of the same *bont* genes/gene clusters within different bacterial variants/species indicates that they have evolved independently. The neurotoxin genes are associated with one of two toxin gene cluster types containing either hemagglutinin (*ha*) genes or *orfX* genes. These genes may be located within the chromosome or extrachromosomal elements such as large plasmids. Although BoNT-producing *C parabotulinum* bacteria are distributed globally, they are more ubiquitous in certain specific geographic regions. Notably, northern hemisphere strains primarily contain *ha* gene clusters while southern hemisphere strains have a preponderance of *orfX* gene clusters. *OrfX C. parabotulinum* strains constitute a subset of this species that contain highly conserved *bont* gene clusters having a diverse range of *bont* genes. While much has been written about strains with *ha* gene clusters, less attention has been devoted to those with *orfX* gene clusters. The recent sequencing of 28 *orfX C. parabotulinum* strains and the availability of an additional 91 strains for analysis provides an opportunity to compare genomic relationships and identify unique toxin gene cluster characteristics and locations within this species subset in depth. The mechanisms behind the independent processes of bacteria evolution and generation of toxin diversity are explored through the examination of bacterial relationships relating to source locations and evidence of horizontal transfer of genetic material among different bacterial variants, particularly concerning *bont* gene clusters. Analysis of the content and locations of the *bont* gene clusters offers insights into common mechanisms of genetic transfer, chromosomal integration, and development of diversity among these genes.

## Introduction

Botulinum neurotoxins (BoNTs) are a worldwide public health issue and are listed as Tier 1 Select Agents due to their potential to pose a severe threat to human and animal health (Federal Select Agent Program Select Agents and Toxins List)^1^. The recent availability of genomes from over 250 BoNT-producing clostridial strains has provided new opportunities to gain perspective about the toxins and their toxin gene clusters, and the relationships of the strains that produce these toxins (Giordani et al., 2015; Mazuet et al., 2016; Williamson et al., 2016).

The neurotoxins are extremely diverse. They have historically been categorized according to the neutralizing ability of serotype-specific antisera (Peck et al., 2017). Seven toxin types (A-G) have been determined using these methods. Four of these serotypes, BoNT/A, BoNT/B, BoNT/E, and BoNT/F, have been definitively linked with human botulism. While these toxins show commonalities in protein structure and activity, genetic sequencing of the toxin serotypes has revealed that they differ by 35–70% in amino acid sequence. A second level of diversity is seen within these toxins; over 40 toxin subtypes have been currently identified having amino acid sequence differences of 2–36% (Peck et al., 2017). The subtypes are labeled with a number following the toxin type, such as BoNT/A1 or BoNT/F5. This diversity among BoNT proteins contrasts with tetanus toxin, (TeNT), which is closely related to the BoNTs in structure and mechanism of action but differs in both its singularity and a lack of non-toxic complex proteins.

The BoNT-producing bacteria are also diverse in their composition. Historically any bacteria that produced BoNTs was considered to be the species “*Clostridium botulinum*” based on the single characteristic of their neurotoxicity, and various species were instead differentiated into four Groups (I-IV) based on their biochemical and metabolic properties. In 1988, Group IV organisms were genetically confirmed to be the species *Clostridium argentinense* (Suen et al., 1988a) and additional BoNT-producing strains were identified and genetically confirmed to be members of *Clostridium baratii* and C*lostridium butyricum* (Suen et al., 1988b). This created a confusing hybrid nomenclature system involving both Group and species designations. However, genomic studies have now confirmed that BoNT-producing strains are represented in seven distinct species (Smith et al., 2018). Within this manuscript the bacteria will be designated by Latin binomials: *C. parabotulinum* (proteolytic *C. botulinum* Group I*)* (Seddon, 1922; Meyer and Gunnison, 1929); *C. botulinum* (non-proteolytic Group II) (Smith, 1977); *C. novyi sensu lato* (*C. botulinum* Group III) (Skarin et al., 2011); BoNT/G-producing *C. argentinense*; BoNT/F-producing *C. baratii*; BoNT/E-producing *C. butyricum*; and BoNT/B-producing *C. sporogenes* (Weigand et al., 2015; Williamson et al., 2016). Examples of both BoNT-producing and non-neurotoxigenic bacteria are found within each species. Table 1 lists information on the species, including the toxin types that they produce.

**TABLE 1:** A listing of BoNT-producing species with Group nomenclature.

Species	Group designation	Toxins produced	Toxin gene cluster type(s)
*C. parabotulinum*	Group I	A, B, F	*orfX* + , *ha* +
*C. sporogenes*	Group I	B	*ha* +
*C. botulinum*	Group II	B, E, F	*orfX* + , *ha* +
*C. novyi sensu lato*	Group III	C, D	*ha* +
*C. argentinense*	Group IV	G	*ha* +
*C. baratii*	Group V	F	*orfX* +
*C. butyricum*	Group VI	E	*orfX* +

The discovery and study of BoNT-producing clostridia has a history spanning more than 100 years (van Ermengem, 1897; Landmann, 1904; Leuchs, 1910; Burke, 1919). *C. parabotulinum* bacteria are the most studied BoNT-producing species, as they produce toxins that are responsible for the vast majority of human botulism cases worldwide. They are most closely related to *C. sporogenes* (Hatheway, 1993; Collins and East, 1998; Stackebrandt et al., 1999), prompting some to consider them as variants of the same species. However, recent studies have indicated that they should be considered separate species (Weigand et al., 2015; Williamson et al., 2016).

The neurotoxins are naturally found in association with several non-toxic accessory proteins, known collectively as the toxin complex. The genes encoding these proteins are arranged in a cluster adjacent to the neurotoxin gene. There are two types of *bont* gene clusters, known as *ha* or *orfX*. The toxin gene clusters encode the neurotoxin protein and a variety of non-toxic proteins. Non-toxin/non-hemagglutinin (NTNH) proteins, expressed by the *ntnh* gene, are universally present in both types of toxin complexes. The *ha* gene clusters produce hemagglutinin proteins (HA70, HA17, and HA33) which form a complex that is directly linked to the NTNH protein. Within the *orfX* gene clusters are three open reading frames (*orfX1, orfX2, orfX3*) that have been shown to produce proteins that apparently form somewhat fragile toxin complexes (Mazuet et al., 2012; Lin et al., 2015; Kalb et al., 2017). The functions of these proteins are not presently known. Other genes that are found within the *bont* gene cluster include *botR*, a transcriptional regulator, and *p47*, whose protein was recently shown to share a structural domain with the OrfX2 and OrfX3 proteins and that is related to the TULIP family of lipid-binding proteins (Gustafsson et al., 2017; Lam et al., 2018).

Each botulinum neurotoxin gene (*bont*) is part of one or the other of the above gene clusters. For example, *bont/B* genes are always found within *ha* clusters, while *bont/E* genes are always associated with *orfX* gene clusters. The *bont* genes within BoNT/A1 strains are unique in that they can be located within either of the two gene clusters. The *bont* genes in BoNT/A5 strains, most BoNT/A1 strains, and all BoNT/B strains are located within the smaller (11.7 kb) *ha* toxin gene cluster (Collins and East, 1998; Carter et al., 2011), while a few *bont/A1* genes, as well as *bont/A2-A4* and *bont/A6-A8* genes, and all *bont/F* genes are located within the larger (∼17 kb) *orfX* gene cluster (Luquez et al., 2005; Hill et al., 2009; Kull et al., 2015; Mazuet et al., 2016). In bivalent strains that express two toxins, such as BoNT/A2B5, BoNT/A1(B), and BoNT/B5F2 strains, both toxin gene clusters may be found within a single bacterial isolate. In those cases, the *bont/A* or *bont*/*F* genes are always within *orfX* gene clusters and the *bont/B* genes are within *ha* gene clusters (Hill et al., 2009; Kalb et al., 2017). The *orfX* gene clusters show a high degree of relatedness which contrasts with the variability of the associated toxin type or subtype. For example, gene clusters with identical or nearly identical *orfX*, *botR*, *p47*, and *ntnh* genes may contain *bont* genes with only ∼60% nucleotide identity.

*C. parabotulinum* strains are commonly isolated from environmental and clinical samples, particularly in association with human botulism cases. While *C. parabotulinum* strains are globally distributed, the distinct predominance of *C. parabotulinum* strains with *orfX* gene clusters in the southern hemisphere and, conversely, the predominance of *C. parabotulinum* having *ha* gene clusters in the northern hemisphere suggests a differential evolution of these bacteria and their toxin genes which may have been influenced by environmental factors.

Currently the genomes of over 150 *C. parabotulinum* strains have been publicly posted in the NCBI database, which provides an opportunity to examine genomic relationships and toxin gene cluster commonalities on a broad scale. An additional 28 *orfX C. parabotulinum* isolates that are mainly located in the southern hemisphere were sequenced and analyzed as part of this study. These genomes have provided an opportunity for in depth study of the relationships of this particular species subset that varies in its genomic characteristics, toxin genes and gene clusters, and geographic locations from their previously studied northern hemisphere counterparts.

A comprehensive approach was used that integrates molecular information and phylogeography in order to provide an understanding of these organisms from multiple perspectives, including an examination of the unique components, arrangements, and locations of their *orfX bont* gene clusters.

Genomic relationships among these *orfX C. parabotulinum* strains were analyzed and new insights associated with the components of their toxin gene clusters and associated co-located genes were revealed in this study. The plasmid and chromosomal locations of the *bont* gene clusters in these strains are dependent on both the species (*C. parabotulinum*) and the gene cluster type (*orfX*), and investigation of unique features within these *bont* gene clusters and surrounding genes suggests connections between the locations of these genes and the processes involved in their acquisition and chromosomal integration.

## Materials and Methods

Source attributions of BoNT-producing clostridia have become more important as we seek to understand links between these bacteria and their effects on humans, domestic animals, and the environment. Toxin subtypes differ in their sensitivities to treatments, and a knowledge of the prevalent toxin types in various geographical regions is key to prevention and treatment strategies. An extensive review of the literature, to include over 60 journal articles and book chapters, was undertaken to provide information relating to the geographic diversity of BoNT/A and BoNT/F producing strains, toxin subtypes, and locations. NCBI records associated with BoNT-producing clostridia and unpublished records from various collections were also examined for sources related to additional strains.

Whole genome sequencing of the 28 newly sequenced isolates was conducted on Illumina MiSeq or GAIIx platforms. Briefly, genomes were assembled with a pipeline that incorporates Trimmomatic (v0.30) (Bolger et al., 2014), BayesHammer (Nikolenko et al., 2013), SPAdes (v3.7.1) (Bankevich et al., 2012), and Pilon 1.17 (Walker et al., 2014). Contaminating contigs were identified with BLAST (Altschul et al., 1990) alignments against known contaminants in the NCBI nucleotide database. Any contig associated with contamination or containing anomalously low average coverage was removed. Closely-related, publicly-available genome assemblies and sequencing read data were downloaded from the National Center for Biotechnology Information (NCBI). If only sequencing read data were available for a genome, an assembly was generated with the approach described above or with SPAdes only. Genome assemblies were annotated with Prokka v1.11 (Seemann, 2014). Genomic data has been deposited in the appropriate NCBI databases. Supplementary Table 1 provides information on all genomes in the study, including NCBI accession information, genome assembly statistics, and average nucleotide identity estimated with MASH (v2.2) (Ondov et al., 2016) compared to a reference genome (*C. parabotulinum* Kyoto-F – GCF_000022765.1).

Core-genome single nucleotide polymorphisms (SNPs) were called as described in Williamson et al. (2017). Sequencing reads were aligned to a reference genome, *C. parabotulinum* Kyoto-F (GCF_000022765.1), with BWA-MEM (v0.7.7) (Li, 2013) and SNPs were called with the Unified Genotyper method in GATK (v3.3) (McKenna et al., 2010; DePristo et al., 2011) within NASP (Sahl et al., 2016). If sequence reads were not available for a genome, reads were simulated from publicly-available genome assemblies with ART (MountRainier) (-ss MSv3 -l 250 -f 75 -m 300 -s 30) (Huang et al., 2012) in order to compare a uniform data type. SNPs were removed from the analysis if the depth of coverage was less than ten or if the allele proportion was less than 0.9. Duplicated regions of the reference genome were identified by a reference self-alignment with NUCmer (Delcher et al., 2002; Kurtz et al., 2004) and SNPs falling in these regions were filtered from all downstream analysis. Maximum likelihood phylogenies were generated from the resulting SNP matrices (bestsnps) with IQ-TREE (v1.4.4) (Nguyen et al., 2015) using the K3Pu F ASC G4 model. The consistency index and retention index were calculated with Phangorn. Trees were viewed in FigTree^2^.

Gene sequences and associated regions of *orfX* toxin gene clusters were extracted from Prokka output files for further analyses. BLASTn (Altschul et al., 1990) was used to identify and compare individual *bont* genes, toxin cluster genes and surrounding genes and intergenic sequences. The *lycA* genes and *arsC* genes (and pseudogenes) were aligned with MUSCLE (v3.8.31) (Edgar, 2004) and phylogenies were generated with IQ-TREE (v1.5.5) (Nguyen et al., 2015) using the following best-fit models (Kalyaanamoorthy et al., 2017): *lycA* gene sequences – TIM3 F G4, *arsC* gene sequences – TPM2u F R2.

Complete *orfX* BoNT/A and BoNT/F genomes were searched for evidence of bacteriophage DNA sequences using PHASTER (Zhou et al., 2011; Arndt et al., 2016). Complete, incomplete, and questionable phage sequences were located and compared in order to gain a better understanding of their movements and integration into chromosomes.

## Results

### Distributions of BoNT-Producing *C. parabotulinum* Strains

The occurrence of human and animal botulism is directly linked to exposure to BoNTs or BoNT-producing bacteria that are resident in the environment or have been introduced via exported foods or other materials. A knowledge of the predominant BoNT serotypes and subtypes among clostridia resident within specific geographic locations is critical to providing effective treatment options.

Bacteria that contain *bont/A* genes and produce BoNT/A are the most commonly isolated BoNT-producing strains. While the geographic distribution of BoNT/A strains is known to be worldwide (Fernandez, 1994; Williamson et al., 2016), environmental studies, literature reviews, and examinations of records in public databases and private collections have determined that BoNT/A subtypes exhibit specific geographic ranges.

The major BoNT/A subtype in the northern hemisphere is BoNT/A1. BoNT/A1 strains with *ha* gene clusters are widespread, but BoNT/A1 within *orfX* gene clusters are quite rare. Exceptions to this would be BoNT/A1(B) strains that contain the *bont/A1* gene within an *orfX* gene cluster and a truncated *bont/B* gene within a complete *ha* gene cluster. These strains are commonly found within the United States (Dabritz et al., 2014; Raphael et al., 2014), and appear to be emerging pathogens in Japan (Kenri et al., 2014) and France (Mazuet et al., 2016).

In contrast, the predominant BoNT/A subtype in the southern hemisphere is BoNT/A2. BoNT/A2-producing *C. parabotulinum* are commonly found in the soils in Argentina (Luquez et al., 2005) and BoNT/A2 is the major causative agent in infant and foodborne botulism there (Sagua et al., 2009). Bacteria producing this toxin type are also found in Australia (McCallum et al., 2015) and they been associated with botulism cases in Africa (Mackay-Scollay, 1958; Smith et al., 1979; Frean et al., 2004; Luquez et al., 2012; Viray et al., 2014). Some *C. parabotulinum* strains are bivalent and contain *bont/B6*, *bont/F4*, or *bont/F5* genes in addition to *bont/A2* genes.

The first BoNT/A3-producing strain, known as the Loch Maree strain, was identified in 1922 in association with a foodborne botulism outbreak in Scotland (Leighton, 1922) and was considered to be unique. However, additional BoNT/A3 strains were isolated decades later in Argentina (Luquez et al., 2012) and recently there was an isolation of a BoNT/A3 strain in connection with a foodborne case in Slovakia (Mad’arova et al., 2017). *C. parabotulinum* strains containing *bont/A4*, *bont/A5*, *bont/A6*, *bont/A7*, and *bont/A8* genes are all quite rare and have the commonality of source locations in the northern hemisphere.

The first BoNT/F-producing strain was isolated in Denmark in 1960 (Moller and Scheibel, 1960) and since then eight distinct BoNT/F subtypes have been identified. BoNT/F strains are comparatively rare and are associated with a variety of clostridial strains, including *C. parabotulinum* (BoNT/F1-F5 and BoNT/F8), *C. botulinum* (BoNT/F6), and *C. baratii* bacteria (BoNT/F7). They have a global distribution, with occasional strains being isolated in North and South America, and across Eurasia from Denmark and Italy to China. *C. parabotulinum* strains producing BoNT/F3, BoNT/F4, and BoNT/F5 have only been isolated from Argentina, while BoNT/F1, BoNT/F2 and BoNT/F8 producers are confined in location to the northern hemisphere.

### Genomic Diversity of OrfX BoNT/A and BoNT/F Strains

Phylogenies based on multiple genomic analysis methods support earlier findings that several clostridial species are capable of harboring *bont* genes and producing BoNTs (Popoff, 1995; Collins and East, 1998; Hill et al., 2009; Weigand et al., 2015; Williamson et al., 2016). One of these species, *C. parabotulinum*, includes a range of genomic variants. Strains containing *ha* and *orfX bont* gene clusters are dispersed throughout the *C. parabotulinum* phylogeny (Williamson et al., 2016). The genomes of 28 strains containing *bont/A* and/or *bont/F* genes within *orfX* gene clusters were sequenced as part of this study, and compared with 91 additional OrfX *C. parabotulinum* genomes using a core genome SNP phylogeny (consistency index with only parsimony informative SNPs – 0.43, retention index – 0.90) that was generated from an alignment of 119,955 SNP positions called from a core alignment of 1,714,645 positions. The core genome SNP analysis did not include SNPs within the toxin cluster genes or within plasmids, as they are not conserved across all genomes.

**FIGURE 1:**
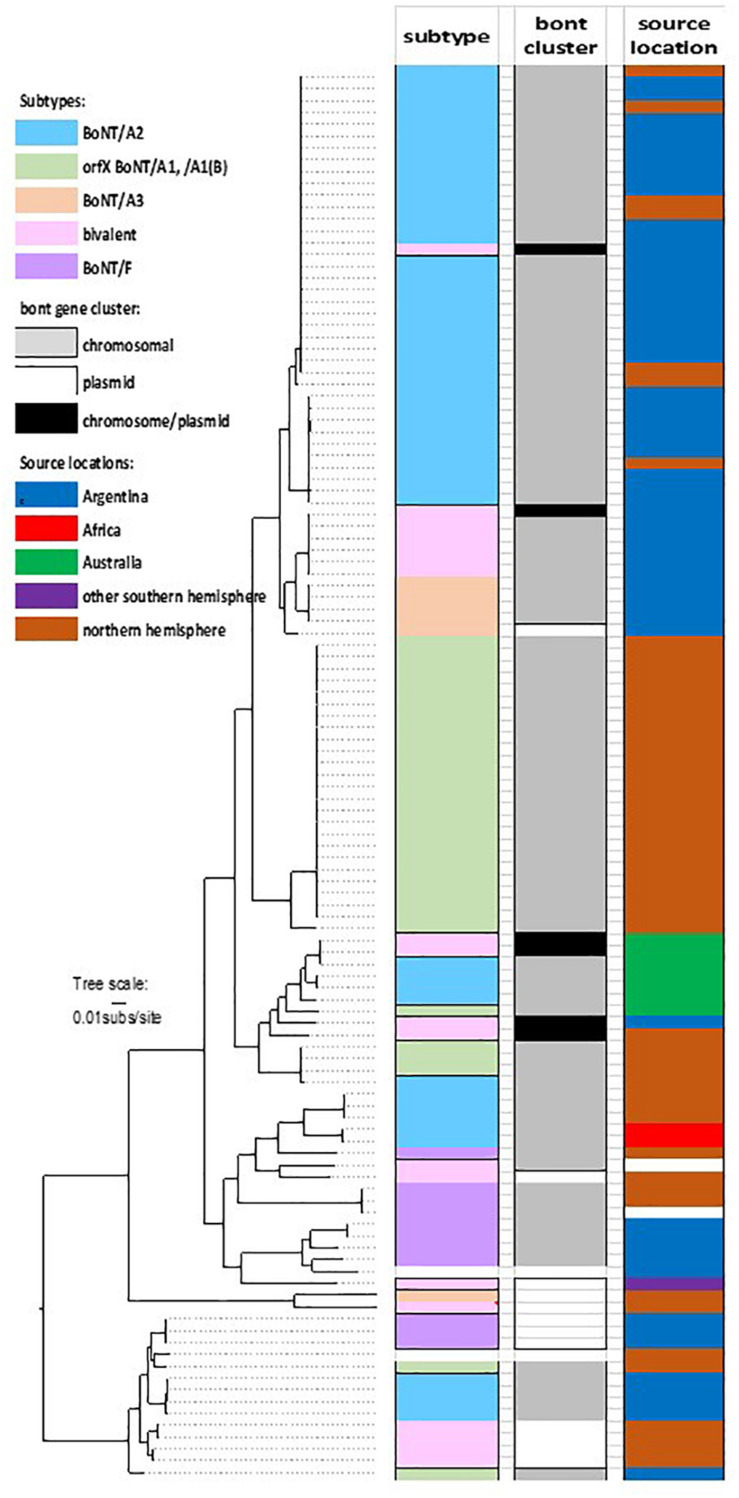
Core genome SNP phylogeny of *C. parabotulinum* strains with *orfX* toxin clusters. The *bont* gene types, gene cluster locations, and geographic locations of the strains are color coded according to the legend. Additional information is listed in Supplementary Table 1 with the genomes in the order they appear in this figure.

Examination of the core genome SNP phylogeny identified several defined clades within the *orfX C. parabotulinum* strains (Figure 1). One of these is a conserved clade composed of subclades containing closely related genomes from strains that were isolated from soils or foodborne botulism cases in northwest Argentina, and a few European strains. Within this major clade are three subclades: one subclade containing 37 genomes having *bont/A2* gene clusters, one subclade with six genomes having *bont/A2* and *bont/F4* gene clusters, and one subclade with five genomes containing *bont/A3* gene clusters. Notably, while BoNT/A2F4 strains form a highly conserved clade that is closely related to Argentinean BoNT/A2 and BoNT/A3 strains, the bacteria that contain only *bont/F4* genes are in a distinct, unrelated cluster of clades. Among these 48 strains, 41 were isolated from a specific region in Argentina. The close relationship of these lineages coupled with their common geographic location is an indication that these strains may have evolved from a single ancestral strain and subsequently acquired different toxin genes. The *bont* genes in these BoNT/A2 strains and several of the BoNT/A3 strains are located within the chromosome, which provides for a greater genetic stability than genes that are within extrachromosomal elements.

While one of the BoNT/A2-producing strains represented in this clade (Kyoto-F) was isolated from an infant botulism case in Japan, it is known that this case was associated with the ingestion of honey and that the honey was likely imported from Argentina (personal communication, Dr. Shunji Kozaki), providing a geographic link between these strains.

Two separate conserved clades that contain genomes of BoNT/A1(B) strains also exhibit localized geographic ranges. For example, in the largest clade of BoNT/A1(B) isolates 19 of 24 strains were located in the United States, four were from Japan and one was from Ecuador, while the smaller clade contained genomes from one Italian and three Japanese strains. With one exception, the strains within the two BoNT/A1(B) clades were isolated in the northern hemisphere.

A more variable clade contains Australian BoNT/A2 strains and one BoNT/A2B6 strain. While the *bont/A2* genes in these strains are located within the chromosome, *bont/B6* genes are universally located within large conjugative plasmids in both *C. parabotulinum* and *C. sporogenes* strains. The existence of *bont/B6* genes within plasmids in multiple clostridial species illustrates cross-species transfer of toxin genes by introduction via their plasmids.

Two African BoNT/A2 strains are part of a second variable genomic clade that includes Italian BoNT/A2 strains and BoNT/F8 It 357. Two subclades containing BoNT/F1 isolates, and BoNT/F4 strains plus the lone BoNT/F3 isolate indicate a relationship with the African strains. The Mexican isolate containing *bont/A6* and *bont/B1* genes is an outlier within this clade that is somewhat related to the BoNT/F3 and BoNT/F4 strains.

Rare BoNT/A1 strains having *orfX bont* gene clusters are found within one of two clades. One isolate from Australia is part of a clade that contains seven Australian BoNT/A2 strains, while two OrfX BoNT/A1 strains that are part of the “bivalent toxin” clade are from the United States and Argentina. Genomes from BoNT/A3 strains also sort into two clades according to location (Argentina and Scotland). While the Argentinian isolates show a definite relationship with geographically related Argentinean BoNT/A2 strains, the Scottish BoNT/A3 strain shows a relationship with the unusual Italian BoNT/A2B7 It 92 strain.

Genomes containing *bont/A2* genes form part of a final variable clade that also contains genomes of bivalent BoNT/A2B5, BoNT/B5A4, and BoNT/B5F2 strains, as well as several BoNT/F5 strains and the two OrfX BoNT/A1 strains previously mentioned. These strains have a global geographic range; many of its members are sourced from Argentina, but locations also include Sweden, Italy, and the United States. With the exception of the BoNT/A2 and OrfX BoNT/A1 strains in this clade, the toxin genes within these genomes are located within large, highly conserved plasmids.

Within this phylogeny are examples of conserved clades containing geographically co-located strains having the same *bont* genes and gene clusters; conserved clades containing different *bont* genes; and variable clades containing unrelated strains having same *bont* genes. While the conserved clades emphasize an ability for a particular strain to become established in a certain region, the variable clades are indications that global movements of these strains do occur, and that transported strains are capable of exchanging their toxin genes with a variety of strain variants and species. While *bont* gene diversity is facilitated by the movement of extrachromosomal plasmids from strain to strain, stability of the genes may be ensured by subsequent integration into the chromosome. Novel toxins have been formed through homologous recombination events that place new toxin genes or gene fragments into existing *bont* gene clusters and, on a lesser scale, through single nucleotide mutations. A study of the composition and location of *orfX bont* genes and gene clusters aids in the understanding of the processes involved in gene cluster movements and integrations.

### Toxin Gene Clusters and Their Locations

Toxin gene clusters in *C. parabotulinum* strains containing *ha bont/A1*, *bont/B*, and *bont/A5* genes are arranged in the following order: *ha70-ha17-ha33-botR-ntnh-bont.* Gene clusters from all other *C. parabotulinum* strains show the following arrangement: *orfX3-orfX2-orfX1-botR-p47-ntnh-bont*. Adjacent genes include the *lycA* gene and, in some cases, *arsC* genes.

*OrfX bont* gene clusters may be located within large, highly conserved plasmids or they may be present as pathogenicity islands (PAIs) within the chromosome. PAIs are gene clusters linked to virulence factors that endow the bacteria with pathogenic properties. They are derived from mobile genetic elements, such as conjugative plasmids or bacteriophage, and are subsequently integrated into the chromosome (Davis and Waldor, 2002). Within the chromosome, PAIs are typically present as discreet genetic units of 10–100 kb distinguished by a lower G + C content than the surrounding genomic DNA. They are often flanked by direct repeat sequences and mobility genes, such as IS elements, integrases, and transposases (Hacker et al., 1997). These general PAI characteristics are in agreement with those of *bont* gene clusters.

**FIGURE 2:**
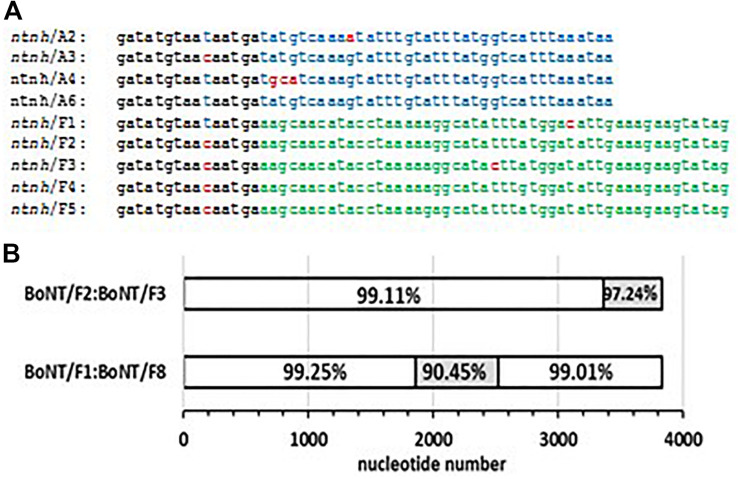
Homologous recombination (HR) events that have exchanged or altered *bont* genes/gene clusters, resulting in the generation of diverse toxin subtypes. **(A)** C-terminal sequence of selected *orfX ntnh* genes showing homologous and disparate sequences that illustrate the recombination event placing *bont/A2/A3* genes within *bont/F* gene clusters, or vice versa. Sequences showing complete identity are in black font, *bont/A*-specific sequences are in blue font, *bont/F* sequences are in green font, and single mutational differences are shown in red font. **(B)** Comparisons of *bont/F2:bont/F3* and *bont/F1:bont/F8* gene sequences showing areas of identity, possibly gained through HR events, and regions where sequences differ (shaded in gray).

It is interesting to note that the non-toxic accessory genes within *orfX* toxin clusters show remarkable conservation, despite their association with multiple *bont/A* and *bont/F* genes. A discontiguous BLASTn comparison of the entire toxin gene cluster sequence (minus the toxin gene) from the BoNT/F1 Langeland strain results in 94–100% identity with gene clusters from over 60 strains located worldwide that contain *bont/A2*, *bont/A3*, *bont/HA*, *bont/F1*, *bont/F3*, *bont/F4*, and *bont/F5* genes. This high degree of toxin gene cluster identity ends at the terminal 50 nucleotides of the *ntnh* gene, where a homologous recombination (HR) event has placed *bont/A* neurotoxin genes within the *bont/F* gene cluster, or vice versa (Figure 2A). This is similar to a recombination event described within the *ntnh* gene that inserted the *bont/A1* gene into the *ha bont/B* toxin gene cluster, producing the *ha bont/A1* gene cluster (Hill et al., 2009).

HR events within *bont* genes have also been responsible for the generation of novel toxin subtypes. It is known that the *bont/A2* gene is a mosaic of the *bont/A1* and *bont/A3* genes (Hill et al., 2007), and comparisons of *bont/F* genes as part of this study has revealed HR events among these subtypes as well. The *bont/F2* and *bont/F3* genes show greater than 99% identity until the final 470 nucleotides, where the identity decreases to 97.24%, indicating an HR event has occurred at its 3’ terminus. Similarly, the *bont/F1* and *bont/F8* genes show >99% identity throughout the 5’ half of the gene and also at final third of its nucleotide sequence; however, between these closely related DNA sections the percent identity decreases to slightly more than 90%, again signaling an HR event has occurred (Figure 2B).

**FIGURE 3:**
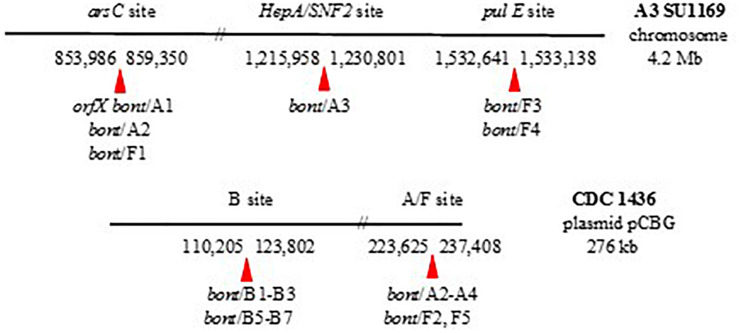
Chromosomal and plasmid *C. parabotulinum orfX bont* gene cluster locations. The sites are numbered according to their base pair location within the BoNT/A3 SU1169 genome and plasmid pCBG from BoNT/A2B5 CDC 1436.

The *orfX bont* gene clusters are found at four distinct genomic locations – three sites within the chromosome and one within plasmids (Figure 3). The locations are identified by their proximity to specific genes. Chromosomally located *orfX bont/A1*, *bont/A2, bont/F1*, and *bont/F8* gene clusters are co-located with the *ars* operon, while *bont/F3* and *bont/F4* gene clusters are inserted between fragments of a split *pulE* gene (Dover et al., 2013; Smith et al., 2020). Plasmid-borne *orfX bont* gene clusters and chromosomal *bont/A3* gene clusters are universally adjacent to *HepA/SNF2*, thermonuclease, and DNA helicase genes.

### The Presence of the *lycA* Gene in *orfX C. parabotulinum* Gene Clusters

The *lycA* gene, while not historically considered a part of the toxin gene cluster, is universally situated alongside *orfX* gene clusters. It is found in two locations: 1) following the *bont* gene with chromosomally located *orfX* toxin gene clusters that are co-located with the *ars* operon and 2) prior to the *orfX3* gene within plasmid-borne toxin gene clusters and chromosomally located *bont/A3*, *bont/F3* and *bont/F4* toxin gene clusters, where the *ars* operon is remotely located.

**FIGURE 4:**
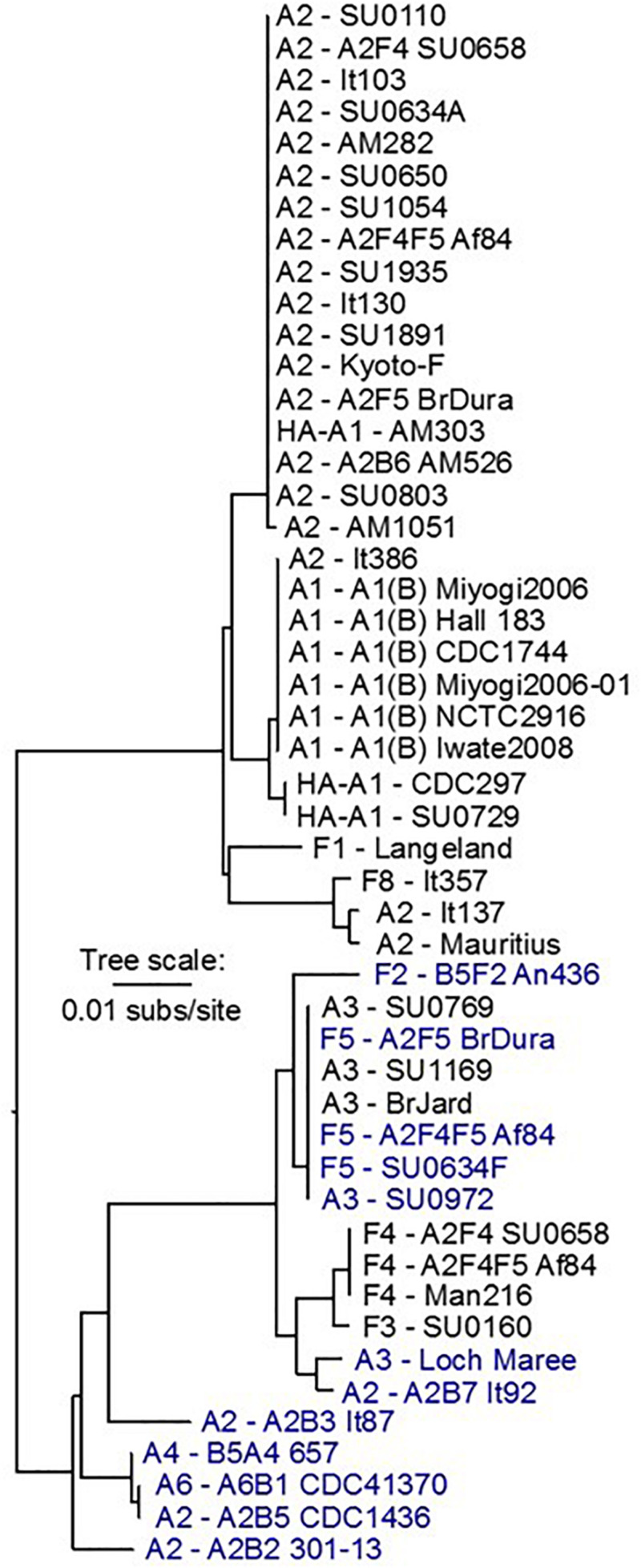
Dendrogram showing the relationship between *bont/A* and *bont/F lycA* gene sequences located within the chromosome and plasmids. Chromosomally located *lycA* genes are in black font and plasmid-borne genes are in blue font. Two major *lycA* variants with nucleotide differences of ∼6% can be discerned. The tree is midpoint rooted.

The *lycA* genes are highly conserved, with two basic variants that correspond to their locations relative to the *bont* and *ars* gene clusters, and are also generally linked to their chromosomal or plasmid location. A phylogeny comparing *lycA* gene sequences (Figure 4) illustrates these relationships. Nucleotide differences between the two *lycA* gene variants are approximately 6%, while differences of less than 2% are seen within the two variants.

The *lycA* gene product is a lysozyme that is homologous to lytic proteins found in *Lactobacillus* and *Streptococcus pneumoniae* bacteriophage (Henderson et al., 1977). These bacteriophage proteins lyse the bacterial cell walls, releasing phage during their lytic cycles. In similar fashion, the *lycA* lysozyme may be responsible for bacterial autolysis and subsequent release of toxin in proteolytic *C. parabotulinum* bacteria (Bonventre and Kempe, 1960; Dineen et al., 2002).

It should be noted that intact *lycA* genes are only found among *orfX* gene clusters from members of *C. parabotulinum*. The *lycA* gene’s ubiquitous presence adjacent to this toxin cluster and the role its protein may play in toxin dissemination suggests that it could be considered a component of *orfX* toxin gene clusters.

### Toxin Gene Clusters Within the Chromosome – Relationships With *ars* Genes

Chromosomally located *orfX bont* gene clusters, with the exception of those containing *bont/A3*, *bont/F3* or *bont/F4*, are co-located with the *ars* operon genes. The *ars* operon encodes proteins that confer resistance against inorganic arsenite that is found in anaerobic environments. The complete *ars* system (*arsR*, *arsD*, *arsA*, *arsB*, *arsC*, *arsM*) is present in most *C. parabotulinum* strains and provides the most efficient removal of arsenic, but operons that are devoid of some *ars* genes appear to provide a minimal level of protection (Lindstrom et al., 2009). The *ars* genes are found both within the chromosome and within mobile extrachromosomal elements in a wide variety of bacteria and are known to be involved in horizontal gene transfer events (Andres and Bertin, 2016). However, with BoNT-producing clostridia, the *ars* operon appears to be strictly located within the chromosome.

**FIGURE 5:**
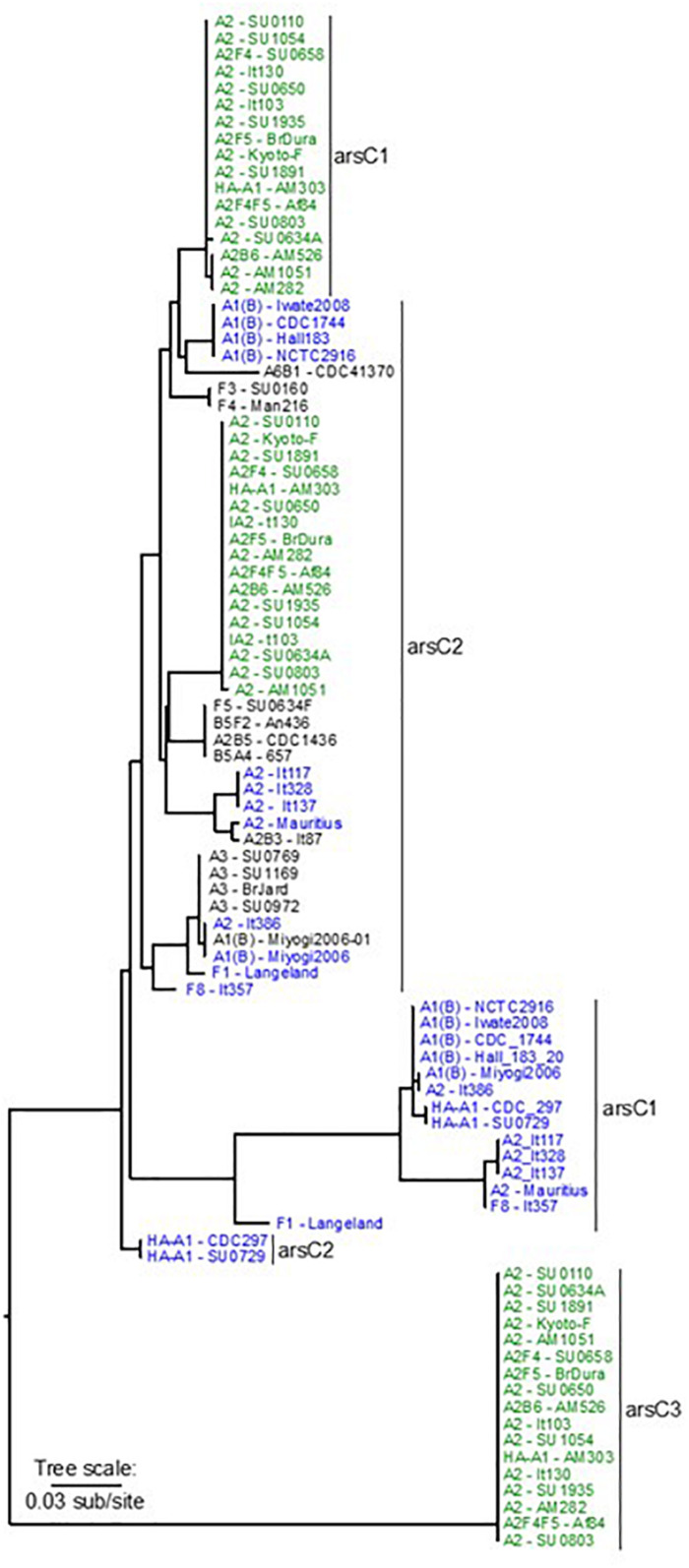
Dendrogram comparing sequences from *arsC1*, *arsC2*, and *arsC3* genes associated with selected BoNT/A and BoNT/F toxin clusters. The *ars* genes are numbered 1, 2, or 3 according to their locations in relation to the *bont* gene cluster. Sequences from strains that contain all three *arsC* genes are shown in green font, sequences in blue font are from strains with *arsC1* and *arsC2* sequences, and sequences in black font are from strains with *arsC2* only. The *arsC3* gene differs by ∼20% in nucleotide residues from *arsC1* and *arsC2*. The tree is midpoint rooted.

Up to three copies of *arsC* genes form part of this operon (Dineen et al., 2004; Hill et al., 2009). The three *arsC* genes are identified here as *arsC1*, *arsC2*, or *arsC3* according to their locations relative to the *bont* and *ars* gene clusters. The *arsC1* gene is adjacent to the *orfX3* gene in the *bont* gene cluster, *arsC2* is found at the beginning of the *ars* gene cluster, and *arsC3*, when present, is located within the *ars* gene cluster. These genes vary in size, ranging from 337 to 392 bp with *arsC1* and 366–417 bp with *arsC2*. The *arsC* genes appear to have deteriorated in some cases, forming pseudogenes. A phylogeny comparing the *arsC1, arsC2*, and *arsC3* genes shows that the *arsC1* genes and the *arsC2* genes are each located within two distinct clades, while the *arsC3* genes form a single clade (Figure 5).

**FIGURE 6:**
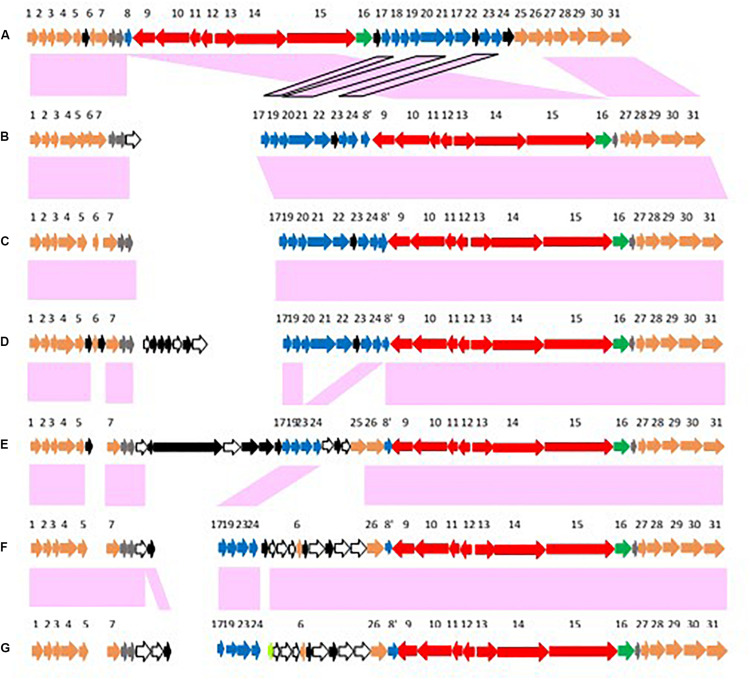
Arrangements of conserved genes surrounding chromosomally located *bont* gene clusters that are adjacent to the *ars* operon. Conserved genes are numbered 1–31 with #9–16 representing the *bont* gene cluster (colored red with the *lycA* gene in green) and #17–24 representing the *ars* operon (colored blue). Supplementary Table 2 lists the genes/encoded proteins corresponding to the numbers shown in Figure 6. Conserved genes that flank this location are colored orange; conserved hypothetical genes are gray; and non-conserved hypothetical genes are black. Panel **(A)** represents an arrangement where the *ars* operon is located downstream from the *bont* gene cluster while panels **(B–G)** represent arrangements where the *ars* operon precedes the *bont* gene cluster. Strains representing these arrangements include: **(A)** most Argentinean and Australian BoNT/A2 strains; **(B)** BoNT/F1 strains; **(C)** OrfX BoNT/A1 strains; **(D)** BoNT/A1(B) strains; **(E)** African BoNT/A2 strains; **(F)** Italian BoNT/A2 trains; and **(G)** Italian BoNT/F8 It 357.

All three *arsC* genes are generally observed in strains containing chromosomally located *bont/A2* genes and with *orfX bont/A1* strain AM303. Where all three *arsC* genes are present, each *arsC* variant is nearly identical in sequence and the *arsC1* and *arsC2* genes are closely related, having greater than 96% identity. The *arsC3* gene is less closely related, with ∼80% identity when compared to *arsC1* or *arsC2*. In these genomes the *ars* operon follows the *bont* gene cluster (Figure 6A). However, with *orfX bont/A1* toxin gene clusters in BoNT/A1(B) strains and OrfX BoNT/A1 strains CDC 297 and SU0729; *bont/F1* gene clusters*;* the *bont/F8* gene cluster; and a few *bont/A2* toxin gene clusters the *arsC3* gene is missing and the location has been re-arranged so that the *ars* operon now precedes *arsC1* and the *bont* gene cluster (Figures 6B–G). Genomes that lack *arsC3* genes show greater variability within their *arsC1* and *arsC2* genes and their *arsC1* genes are within a distinct clade that differs by 6–7% from the clade in genomes having three *arsC* variants. In strains that lack both *arsC1* and *arsC3*, such as BoNT/A3, BoNT/F3, and BoNT/F4 strains, *bont* gene clusters are inserted into the chromosome at sites that are remote from the *ars* genes.

While the *bont* genes and gene arrangements immediately surrounding the *bont* gene clusters vary, the same conserved genes (#1–7 and #27–31 in Figure 6) flank these regions, placing them at a common location within the chromosome. Figures 6E–G show arrangements where *arsC1* and arsC2 are present but several other *ars* genes are absent, an indication of diminished arsenic resistance in these strains. Supplementary Table 2 lists the genes/encoded proteins corresponding to the numbers shown in Figure 6.

The re-arrangement of the *bont* gene cluster related to the *ars* operon indicates an evolutionary shift, as does the loss of several *ars* genes seen with the arrangements of *bont* gene clusters in African and Italian strains. The arrangement in OrfX BoNT/A1 and BoNT/A1(B) strains suggests their *bont/A1* gene clusters may have evolved from a common ancestor, but several distinct genes that are lacking in the OrfX BoNT/A1 strains (Figure 6B) have been inserted upstream from the *bont/A1* gene cluster in the BoNT/A1(B) strains (Figure 6C), indicating an evolutionary divergence. Similarly, the arrangements in the African and Italian strains (Figures 6D,E, respectively) show a clear ancestral relationship, but additional genes have been inserted between the *ars* and *bont* cluster genes in the Italian strains, again signaling an evolutionary divergence. While these *bont* gene clusters show relationships within their non-toxin *bont* cluster genes and chromosomal integration arrangements, the strains that harbor these genes are often unrelated, emphasizing the differential evolution of the bacteria and the *bont* genes.

### Involvement of the *pulE* Gene in the Integration of *bon*t/F Gene Clusters Into the Chromosome

**FIGURE 7:**
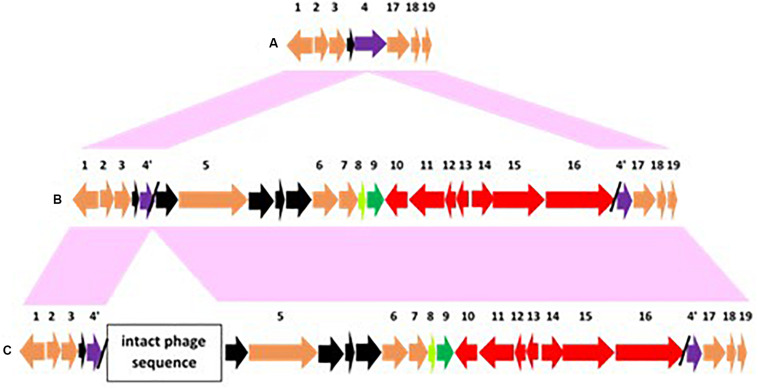
Illustration of the integration location of chromosomally located *bont/F3* and *bont/F4* cluster genes, which is distinct from the *ars* operon location. At this site the *bont* gene clusters in panels **(B,C)** (#9–16, shown in red with the *lycA* gene in green) are inserted within a split *pulE* gene (#4 and 4’, purple). Supplementary Table 3 lists the genes/encoded proteins corresponding to the numbers shown in Figure 7. Additional conserved known genes are colored orange and conserved hypothetical genes are in black. Transposase genes are in light green. **(A)** Insertion region in BoNT F5 BrDura showing a complete *pulE* gene and an absence of the *bont* gene cluster. **(B)** The same region in BoNT F4 SU1425, showing an approximately 35 kb region between the truncated segments of the *pulE* gene. **(C)** The 92.5 kb DNA sequence in BoNT F3 SU0160 with the identical sequence found in BoNT F4 plus an inserted segment containing an intact bacteriophage.

As *bont/F3* and *bont/F4* gene clusters are remotely located (∼250 kb – 1.1 mb) from *ars* operons that lack both *arsC1* and *arsC3* genes, the chromosomal integration process differs from strains that contain *bont/A2* and *bont/F1* gene clusters. The insertion process for the *bont/F3* and *bont/F4* gene clusters involves excision of a *pulE* gene with insertion of *bont* gene cluster DNA within the *pulE* gene (Figure 7; Dover et al., 2013; Smith et al., 2020). The *pulE* gene is one of 15 genes that encode the *pul* secreton, which is type II secretion system for proteases and toxins in gram negative bacteria. This mechanism is similar to the insertion mechanisms for *bont/E* gene clusters that involve a split *rarA* gene (Hill et al., 2009) and *bont/F6* genes, where the split gene is *topB* (Carter et al., 2013); however, while a second intact *rarA* or *topB* gene is seen in these insertions, there is no duplicate intact *pulE* gene adjacent to the inserted *bont/F3* or *bont/F4* gene clusters. In clostridia the *pulE* gene may be non-essential, as most of the remaining genes encoding proteins that form this secretion system are absent. Figure 7 shows the arrangement of the genes present within and flanking the split *pulE* gene for BoNT F3 SU0160 and multiple BoNT F4 strains, and the intact *pulE* gene in BoNT F5 strains, where the *bont* gene cluster resides within a plasmid. With both the *bont/F3* and *bont/F4* gene clusters the inserted genetic material between the two *pulE* gene fragments contains, in addition to the *bont* gene cluster, a 35 kb DNA fragment that includes a PHP domain protein gene, a KAP family p-loop domain protein gene, a helix-turn-helix protein gene (#5–7 in Figures 7B,C), and several hypothetical proteins. A complete listing of the genes/encoded proteins numbered in Figure 7 is shown in Supplementary Table 3. However, with the *bont/F3* gene cluster, an additional 57 kb DNA fragment encoding an intact bacteriophage is present (Smith et al., 2020). The significance of this finding is as yet unknown. An interesting finding is a similar split *pulE* gene within the BoNT/A3 Loch Maree strain which is devoid of *bont* cluster genes but instead contains an intact prophage sequence that is distinct from the one associated with the *bont/F3* gene cluster (Dover et al., 2013; Smith et al., 2020), marking this as a general site for integration of prophage sequences as well as *bont* gene clusters.

### Toxin Gene Clusters Within Plasmids

While most of the bacterial strains analyzed here contain *bont* genes within the chromosome, several strains contain one or more *bont* genes within large (∼250–280 kb) conserved conjugative plasmids. Characteristics of the plasmids for which complete sequence information is known are shown in Table 2. The strains that harbor plasmid-borne *bont* genes are relatively rare, contain a wider variety of *orfX bont/A* and *bont/F* genes, and are found in genomes that are within variable phylogenetic clades. Strains containing these plasmids have been isolated in North and South America, Europe, Asia, and Australia, indicating an ease with which these plasmids may spread among globally located strains. The ability of these plasmids to move between different clostridial strains and species has also been confirmed through laboratory experiments (Marshall et al., 2010).

**TABLE 2:** Characteristics of plasmids from *C. parabotulinum* containing *bont/A* and/or *bont/F* genes.

BoNT subtype(s)	Strain	Plasmid ID	Accession #	Size (bp)	Toxin cluster location
A2B3	It 87	p1_A2B3_87	AUZB01000012.1	275,568	Plasmid/plasmid
A2b5	CDC 1436	pCBG	CP006909.1	275,986	Plasmid/plasmid
A2B7	It 92	p_A2B7_92	AUZA01000014.1	260,807	Plasmid/plasmid
A2f4f5	Af84	pCLQ	AOSX01000021.1	246,124	A2, F4 chr; F5 plasmid
A2f5	BrDura	pRSJ20_1	CP014152.1	241,076	A2 chr; F5 plasmid
A3	SU0972		MWIV01000007.1	238,810	Plasmid
A3	Loch Maree	pCLK	NC_010418	266,785	Plasmid
B5a4	657	pCLJ	NC_012654	270,022	Plasmid/plasmid
B5a4	CFSAN034200	p1_CDC51232	CP031095	270,024	Plasmid/plasmid
B5f2	An436		LFON01000008	171,021	Plasmid/plasmid
F5	SU0634F	pRSJ3	CP013710	244,784	Plasmid
F5	SU0632		MWIY01000007.1	243,777	Plasmid

These plasmids may contain one or two toxin gene clusters, located at two distinct sites. One site exclusively contains *ha bont/B* gene clusters and the second site contains *orfX bont/A* or *bont/F* gene clusters (Figure 3). Plasmids that contain only one *bont* gene cluster (*bont/A3*, *bont/F5*, or *bont/B* gene clusters) are known as well as those with two *bont* gene clusters (*bont/F2* plus *bont/B5* genes or *bont/A2, bont/A4*, or *bont/A6* paired with *bont/B* genes). A search for plasmid gene sequences within *C. parabotulinum* using BLASTn indicates that, with one exception, all of these related large *C. parabotulinum* plasmids contain *bont* genes and, because of their common toxin cluster locations within the plasmid, none contain both *bont/A* and *bont/F* gene clusters together.

Identical *bont* genes and gene clusters have been located either within plasmids or the chromosome of *C. parabotulinum* strains, which confirms the ability of these plasmid-borne genes to integrate into the chromosome and also affords us the opportunity to study the extent of exchanged genetic material. This duplicate location of *bont* clusters in the chromosome or plasmids is not specific to *orfX C. parabotulinum* strains. Gene clusters containing *bont/B1* or *bont/B2* genes have been identified that are located within either the chromosome or plasmids (Franciosa et al., 2009), as well as *bont/E1*, *bont/E3*, and *bont/E10* genes (Zhang et al., 2013; Carter et al., 2016) and *bont*/F7 genes (Halpin et al., 2017; Mazuet et al., 2017).

BLASTn analysis of complete plasmids from twelve of these strains show 95–100% identity over large segments of the plasmid sequence, illustrating the close relationships among them (Table 3). However, they also show evidence of continuing insertion, deletion, and inversion events. For example, the plasmid from strain An436 (LFON01000008) that contains *bont/B5* and *bont/F2* gene clusters is closely related to the strain 657 plasmid (pCLJ) that contains *bont/B5* and *bont/A4*, but a large (99 kb) deletion can be seen and, in addition, the *bont/F2* gene cluster is reversed in orientation compared to the rest of its plasmid DNA (Hill et al., 2009).

**TABLE 3:**
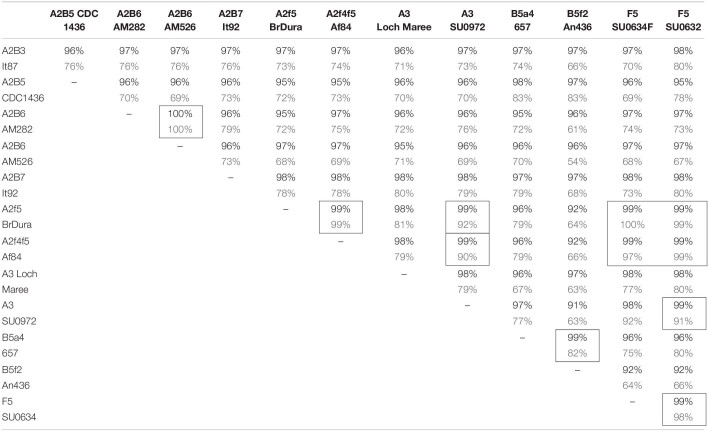
Comparison of large plasmids containing *bont* gene sequences.

*Note that these plasmids share greater than 90% identity over at least 60% of their sequence. The percentage identity is in black font and the percent coverage between the plasmids is in grey font. Closely related plasmids (99–100% identity with >96% coverage), such as those containing bont/B6 or bont/F5 genes, are bordered. The comparisons were generated using discontiguous BLAST analysis (NCBI).*

**FIGURE 8:**
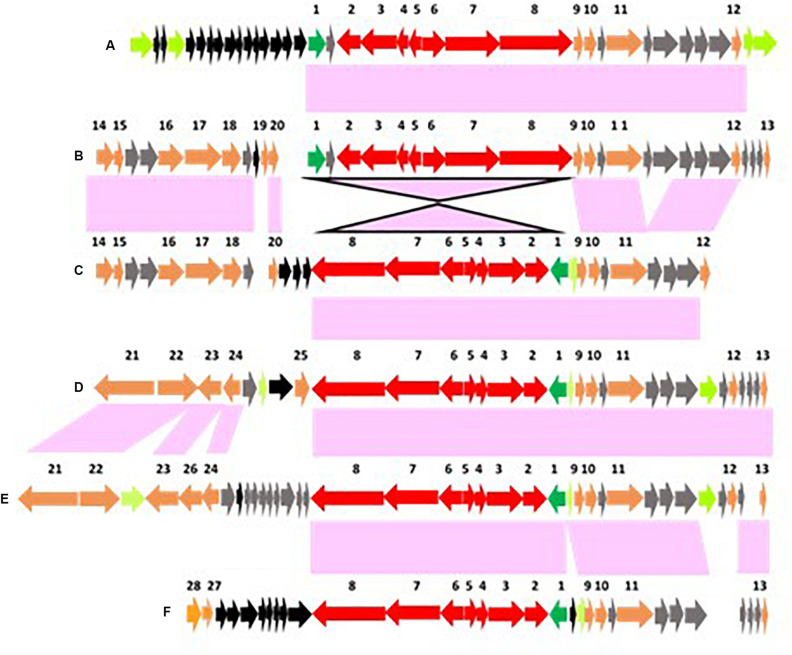
Arrangements of conserved genes surrounding *orfX bont* gene clusters that are located within plasmids. Conserved genes representing the *bont* gene cluster are numbered #1–9, colored red with the *lycA* gene in green. Conserved genes that flank this location are colored orange, including the *HepA/SNF*, *Tnase*, and DNA helicase genes (#9–11); conserved hypothetical genes are gray; and non-conserved hypothetical genes are black. Supplementary Table 4 lists the genes/encoded proteins corresponding to the numbers shown in Figure 8. Panels **(A,B)** illustrate arrangements where the *bont* gene cluster is in the same orientation as the surrounding genes, while panels **(C–F)** show arrangements where the *bont* genes are in opposite orientation. Examples of these arrangements are found with **(A)** the *bont/A2* gene cluster in strain BoNT/A2B3 It 87; **(B)** the *bont/A2* gene cluster in BoNT/A2B5 CDC 1436; **(C)** the *bont/F2* gene cluster in BoNT/B5F2 An436; **(D)** the *bont/A2* gene cluster in BoNT/A2B7 It 92; **(E)** the *bont/F5* gene cluster in BoNT/A2F5 BrDura, BoNT/F5 SU0634F, and BoNT/A2F4F5 AF84; and **(F)** the *bont/A3* gene cluster in BoNT/A3 Loch Maree.

In fact, the orientation of the *bont* gene cluster in relation to the surrounding genes varies from plasmid to plasmid. The *bont/A* gene cluster (#1–8 in Figure 8) in the BoNT/A2B3 It 87 strain, BoNT/A2B5 CDC 1436, BoNT/A6B1 CDC 41370, and BoNT/B5A4 strains is in the same orientation as the surrounding genes (Figures 8A,B), but with BoNT/A2B7 It 92, BoNT/A2F5 or BoNT/F5 strains, and BoNT/A3 Loch Maree the genes are in the opposite orientation from the surrounding genes (Figures 8C–F). The factors that govern the orientation of *bont* gene clusters are not understood; however, the presence of transposes adjacent to the *lycA* gene with most of these gene clusters suggests a possible role for them in gene integration and orientation.

In the plasmid-borne *orfX bont* cluster regions, the gene sequences that are downstream of the *bont* gene cluster are well-conserved, showing that all *orfX bont* clusters are located in the same position within the plasmid, regardless of the *bont* gene present. The *lycA* and *bont* clusters are followed by *HepA/SNF2*, which encodes a DNA helicase protein, plus thermonuclease (*Tnase*), and an additional DNA helicase gene (#9–11 in Figure 8). However, the upstream sequences are arranged in multiple patterns.

The upstream genes in the BoNT A2b3 It 87 strain differ from those in other plasmids and contain over a dozen unique hypothetical genes (Figure 8A). Several transposases flank the upstream and downstream genes. A conserved upstream gene sequence arrangement is seen with BoNT/A2b3 CDC 1436, BoNT/B5a4 657, and BoNT/A6b1 CDC 41370 strains that includes AraC family transcriptional regulators, a methionine adenosyltransferase gene, the chaperone ClpB gene, a cytidine deoxyribosyltransferase gene, and a thiamine biosynthesis protein gene (#14–20 in Figures 8B,C). These strains are geographically related, having been isolated in the southwestern U.S. and Mexico. A similar arrangement is seen in the North American BoNT/B5F2 strains.

A different arrangement is seen in the Italian A2b7 It 92 strain and strains containing bont/*F5* genes where the upstream genes have been replaced with five unique genes (genes encoding a single strand binding protein gene, a DNA polymerase III subunit gene, and A-type inclusion proteins) (#21–24 in Figures 8D,E). Variable numbers of transposase genes and genes encoding hypothetical proteins are also present within these regions.

The upstream region of the plasmid *bont/A3* gene cluster in the Loch Maree strain consists of nine unique hypothetical genes and genes encoding a putative ABC transporter lipoprotein and an adenylate and guanylate cyclase protein (#27 and #28 in Figure 8F). This region is similar to, but not identical with, that of the *bont/A3* gene cluster region in strain SU0972. A complete listing of the numbered genes/encoded proteins in Figure 8 is shown in Supplementary Table 4.

The conserved downstream gene sequences identify a common *orfX bont* gene cluster location within the plasmid but the varied upstream DNA sequences may provide some clues as to why these *bont* gene clusters have not integrated into the chromosome. The *HepA/SNF2*, *Tnase*, and DNA helicase genes are also present in the absence of *orfX bont* gene clusters in plasmids that contain only *ha bont/B* clusters. Their presence may be an indication of the past existence of *orfX bont* gene clusters at that site or an opportunity for possible future acquisitions. Similar to the “chicken or the egg” quandary, it is not definitively known whether these *bont* gene clusters originated within plasmids followed by integration into the chromosome, whether the opposite is true, or whether movements in both directions is possible.

**FIGURE 9:**
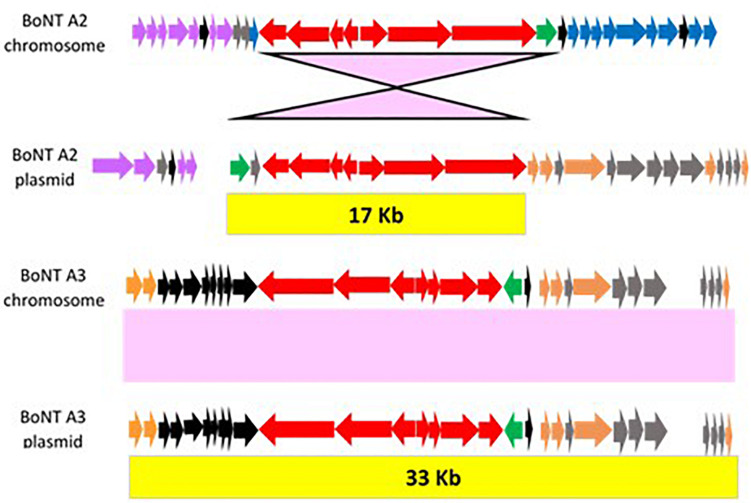
Illustrations of conserved DNA sequences that are shared between chromosomal and plasmid located *bont* clusters. With BoNT A2 strains, conserved sequences comprise only the actual *bont* cluster and *lycA* genes, while the conserved sequence with BoNT A3 strains includes additional surrounding genes. The *bont* cluster genes are colored red, the *lycA* gene is green, and other conserved known genes are orange. Genes that are unique to chromosomal or plasmid-borne *bont/A2* gene clusters are shown in purple. Conserved hypothetical genes that are common to both plasmid-borne *bont/A2* and plasmid and chromosomally located *bont/A3* are in gray and conserved hypothetical genes that are exclusive to *bont/A3* are in black. Specific gene identifications are listed in Supplementary Tables 2, 4 and are related to Figure 6A (*bont/A2* chromosome), 8B (*bont/A2* plasmid), and 8F (*bont/A3* chromosome and plasmid).

An understanding of the gene components and arrangements surrounding identical *bont/A2* and *bont/A3* gene clusters within the chromosome and plasmids presents an opportunity to examine the extent of genetic material that is exchanged within these genetic regions and potentially identify genes or insertion elements that may aid in *bont* gene cluster integration. When chromosomal and plasmid *bont* gene cluster regions of *bont/A2* gene clusters are compared, only the *lycA* and *bont* gene cluster sequences are found to be present in both locations, so that the 17 kb of material transferred between the plasmid and chromosome is strictly composed of the *lycA/bont* gene cluster (Figure 9). In this figure, the *bont* gene cluster genes are colored red and the *lycA* gene is green. Known genes that are unique to each site are colored in purple in Figure 9, while conserved known genes are in orange, conserved hypothetical genes are in gray or black. Individual genes are identified in Supplementary Tables 2,4, as numbered in Figure 6A (BoNT/A2 chromosome) and Figure 8B (BoNT/A2 plasmid).

However, with BoNT/A3 strains the sequence that has been transferred is comprised of a 35 kb highly conserved region surrounding the inverted *bont/A3* gene cluster which includes two known genes and nine conserved hypothetical genes that are located upstream of the *bont/A3* gene cluster, and the downstream *HepA/SNF*, *Tnase*, and DNA helicase genes. Individual genes surrounding the *bont/A3* gene clusters are identified in Supplementary Table 4, as numbered in Figure 8F (BoNT/A3 chromosome and plasmid).

SNF family proteins contain helicase-like domains and often reside within large multi-protein complexes that may facilitate various DNA manipulations (Ryan and Owen-Hughes, 2011). Thus, it is possible that the proteins encoded by the *HepA/SNF2* and DNA helicase genes may function as facilitators for the transfer of the *bont/A3* gene cluster from plasmid to chromosome, or vice versa. While the plasmid-borne *bont/A2* and *bont/A3* gene clusters are located at the same site, these gene clusters are differentially placed at discrete sites within the chromosome implying interaction processes involving distinct target and facilitating genes.

The *bont/A2* gene clusters are commonly found within the chromosome, and only in rare cases are they located within plasmids. As there is an association between chromosomally located *bont*/*A2* gene clusters and *arsC* genes, an examination of the *ars* gene cluster regions within the genomes of several strains having plasmid-borne *bont/A2* gene clusters (BoNT/A2B3 It87, BoNT/A2B5 CDC 1436, and BoNT/A2B7 It92) was undertaken. The chromosomally located *ars* gene operons in these strains contained *arsC2* genes, however, none contained the *arsC1* or *arsC3* genes, which provides an indication by omission of the possible involvement of the *arsC1* genes in the movement of *bont/A2* gene clusters between plasmid and chromosome.

Similarly, the sequence regions surrounding the *bont/A3* gene cluster within the chromosome of BoNT/A3 SU1169 were compared with the same region in BoNT/A3 Loch Maree, where the *bont/A3* gene cluster is found within a large plasmid. The Loch Maree chromosome is missing a 44.7 kb region found within SU1169 that includes the entire ∼35 kb region that is conserved among chromosomal and plasmid-borne *bont/A3* clusters plus additional genes, hinting that the as-yet-unknown facilitators for chromosomal insertion of the *bont*/A3 gene might be located within the 45 kb region that is absent in the Loch Maree strain. Further study of these DNA sequences is needed to enable a better understanding of how their *bont/A3* gene clusters are excised from plasmids and integrated into the chromosome.

### Contributions From Bacteriophage

Both intact and incomplete bacteriophages are commonly found among the BoNT-producing clostridia. Intact active phages have been recovered from mitomycin C-induced lysed cultures from BoNT/A, BoNT/B, BoNT/E, and BoNT/F strains (Eklund et al., 1969). In addition, it is well known that the genes for type C and D toxins are associated with bacteriophage DNA, that “curing” of these bacteria of its phage results in a reversion from toxic to non-toxic status, and that subsequent infection with isolated phage preparations reactivate its toxicity (Eklund and Poysky, 1974). Bacteriophage are also known to be vehicles for the general transfer of PAIs.

Prophage DNA sequences have also been located within the genomes of numerous *orfX C. parabotulinum* organisms. A search for phage sequences in 15 complete *orfX* BoNT/A and BoNT/F genomes using PHASTER (Zhou et al., 2011; Arndt et al., 2016) uncovered more than 19 intact phages within the chromosomes, and numerous incomplete phage sequences were discovered within both the chromosome and the large highly conserved plasmids among these strains. The majority of the identified phages were found within Argentinean BoNT/A2, BoNT/A3, and BoNT/F strains and were related to phages associated with either *Clostridium difficile* or *Clostridium tetani* (Table 4). Some phages associated with *Clostridium sporogenes* and *Clostridium tyrobutyricum* were also identified. The additional finding of shared DNA sequences with prophage fragments from the phage Clostr_c_st (the phage that contains *bont/C* and *bont/D* gene clusters) in the *C. parabotulinum bont*-containing plasmids is not surprising, as these shared sequences were generally linked to non-toxic genes within *bont* gene clusters. However, some conserved transposases were noted as well.

**TABLE 4:** A sampling of bacteriophages found within *orfX* + BoNT/A and BoNT/F *C. parabotulinum* strains.

Phage	Phage type	Associated bacteria	*C. parabotulinum* strains containing intact phage sequences	*C. parabotulinum* strains with partial phage sequences*
phiCT19406A = phiCTC2A	unclassified dsDNA virus	*C. tetani* ATCC 19406 *C. tetani* C2	A2b5 CDC 1436	B5f2 An436
phiCT19406B = phiCT453B	Siphoviridae	*C. tetani* ATCC 19406 *C. tetani* ATCC 453	A2 Kyoto-F F4 SU1425 A2 CDC 53174 A2f5 SU0650 F1 Langeland	A2 SU0994 A2 SU1275 A2 SU0807 A2 SU0801 A2 SU0998 A2 SU0945 A2 SU1072 A2 SU1054
phiCT19406C	unclassified dsDNA virus	*C. tetani* ATCC 19406	A2 Kyoto-F A2 SU1259 A2 SU0634A A2 SU0994 A2b5 CDC 1436 A2 SU1064 A2f5 SU0650 A2 SU1072 A3 Loch Maree A2 SU1054 A3 SU1169 A2 SU1275 A3 CDC 54064 A2 SU1917 F1 Langeland A2 SU1274	224-13 SU1074 SU0807 SU0801
phiC2	Myoviridae	*C. difficile* CD27	F1 Langeland F1 230613 F1 Walls 8G	
phiCD27 = phiMMP02	Myoviridae	*C. difficile* CD27 = *C. difficile* CD119	A2 Kyoto-F A2 SU1259 A2 SU1275 A2 SU1917 A2 SU1274 A2 SU1891 A2 SU1072 A2 SU0807 A2f5 SU0650 A2 SU1064 A2f5 BrDura A2 SU1054	A2 SU0998 A2 SU1937 A2 SU1112 A2 SU0801 A2 SU1887 A2 SU0994 A2 SU1934 A2 SU1074 A2 224-13
phiCD38_2	Siphoviridae	*C. difficile* CD38	A2 Mauritius	
phiCD119 = MMP04	Myoviridae	*C. difficile* CD119 = *C. difficile*	B5a4 657 AB CFSAN034200	CDC 69096
phiCD506		*C. difficile* CD506	A2f5 BrDura	A3 CDC 54064 SU0635W
phiCD6356	Siphoviridae	*C. difficile* CD6356	A2f5 BrDura	A3 SU1169 A3 CDC 54064
phiSM101	Siphoviridae	*C. perfringens* SM101	F1 Langeland F1 230613 F1 Walls 8G	
phi8074_B1	Siphoviridae	*C. sporogenes* ATCC 8074	A2 0634A F5 SU0634F A2b5 CDC 1436 CDC 69096 B5a4 657 CFSAN034200	
phiCTP1	Siphoviridae	*C. tyrobutyricum*	B5a4 657 AB CFSAN034200	
Bacilli_Moonbeam	Myoviridae	*B. megaterium*	F3 SU0160	
Bacill_phIS3501	Siphoviridae	*B. thurgensis v israelensis*	F1 Langeland F1 230613 F1 Walls 8G	
OH2	Siphoviridae	*T. thermophilus* HB8	B5a4 657	

*Bacteria listed as containing partial prophage sequences show >65% coverage with >98% identity using BLAST analysis at the nucleotide level. Some partial sequences may represent overlapping phage sequences.

A bacteriophage associated with *Bacillus megaterium*, Bacilli_Moonbeam, was found adjacent to the *bont/F3* gene cluster in the genome of BoNT/F3 SU0160. This is a unique association of an intact phage sequence with the *bont* cluster of an *orfX C. parabotulinum* strain, as in all other cases complete prophage DNA sequences are remotely located from the *bont* gene cluster. However, it is not known if the *bont/F3* gene cluster is actually part of the prophage, or if they are simply co-located.

Individual genes related to incomplete phage sequences have also been identified that are scattered among the conserved regions surrounding many *bont* clusters within both the chromosome and plasmids. These may represent remnants of ancient phages that previously integrated into the chromosome, possibly in association with *bont* gene clusters, or they may indicate that the present day conjugative plasmids responsible for the movement of *bont* gene clusters may have originated as bacteriophages where certain phage-specific genes have deteriorated while elements necessary for conjugation and *bont* genes have persisted. The significance of these bacteriophages may be underappreciated as contributors to overall genetic diversity in these bacteria through the placement of foreign genes into the chromosome via HGT.

## Discussion

*Clostridium parabotulinum* is a diverse species in their geographic range, in their genomic properties, and in their neurotoxins. BoNT-producing *C. parabotulinum* bacteria have a global distribution but are mainly found in temperate and subarctic regions in both the northern and southern hemispheres. It is noteworthy that, while the majority of *C. parabotulinum* strains isolated in the northern hemisphere harbor *bont* gene clusters containing *ha* genes, in the southern hemisphere the *bont* gene clusters predominantly contain *orfX* genes. As clostridia are spore-forming bacteria, this discrepancy in distribution may be due to differential movements of their bacterial spores. East-west movements among strains containing similar *bont* gene clusters, which could primarily be due to transport of bacterial spores on upper level wind currents, may be more common than north-south movements, which are more likely the results of carriage of spores by migrating birds or due to transfers of contaminated soil, plants, or foodstuffs.

There is evidence that certain geographic areas have provided conditions conducive to the widespread colonization of phylogenetically related bacterial strains, or clonal expansions. However, naturally occurring or manmade movements of bacterial spores from these areas have provided opportunities for these bacteria to become established in faraway regions and allowed for exchanges of novel genetic material between different bacterial strains via horizontal gene transfers (HGT). Comparisons of genomes and *bont* gene cluster sequences reveals that closely related strains have acquired different *bont* genes, often within related *bont* gene clusters, while unrelated strains may contain identical *bont* genes/gene clusters. The closely related strains isolated in northwest Argentina that contain *bont/A2*, *bont/A2F4*, or *bont/A3* genes might be considered clonal expansions, while the finding of phylogenetically diverse *C. parabotulinum* strains located in Argentina, Africa, and Australia that contain identical *bont/A2* gene clusters is an example of the apparent movements of these strains with subsequent exchanges of genetic material among them. Phylogenetic analysis of the genomes representing a subset of *C. parabotulinum* strains that contain *orfX* gene clusters demonstrates these relationships, revealing the presence of several clades that are highly conserved, contrasting with others that are quite variable.

HGT events may be accomplished following active infection by bacteriophage or by movement of genes that are incorporated into mobile genetic elements such as temperate prophage DNA or conjugative plasmids. All three mechanisms may have been utilized in the movement of toxin genes among the BoNT-producing clostridia, but with *C. parabotulinum* strains the majority of such transfers appear to involve conjugative plasmids. However, bacteriophage gene remnants are frequently found adjacent to *bont* gene clusters within the chromosomes and extrachromosomal plasmids. These remnants contain DNA sequences that encode various viral components, including recombinases, integrases, and transposases. It is interesting to speculate that perhaps these large plasmids have originated from bacteriophage that subsequently lost DNA sequences encoding vital structural genes, so that ancient bacteriophages may have evolved to become modern conjugative plasmids.

Once the plasmid-borne *bont* gene clusters have established a presence in a bacterial strain, they may proceed to integrate into their host chromosome and/or exchange genetic material with existing *bont* genes or gene clusters. This is accomplished through homologous recombination (HR) events that involve alignment of paired gene sequences, excision of a particular gene or intergenic sequence, and insertion of a new or exchanged DNA sequence. HR events within *ntnh* genes have placed *orfX bont/A1* genes within *ha bont/B* gene clusters, and *bont/A2* genes within *bont/F1* gene clusters, and they are responsible for some of the toxin subtype diversity seen among *C. parabotulinum* strains. It is known that the *bont/A2* gene resulted from an HR event involving *bont/A1* and *bont/A3* genes, and there is evidence of HR interactions between *bont/F2* and *bont/F3* and *bont/F1* and *bont/F8* that may have shaped their individual genetic identities.

Chromosomal integrations of entire *bont* gene clusters also utilize HR processes, including the necessity for paired genes or intergenic sequences. For example, integrations of *bont/A2* and *bont/F1* gene clusters, that occur at a common site within the chromosome, are dependent on paired *arsC1* and *arsC2* genes. Chromosomal integration provides a more stable location within the bacteria than extrachromosomal locations - it has been shown both experimentally and through natural occurrence that extrachromosomal DNA is subject to deletion through plasmid loss or “curing” of bacteriophage, while loss of chromosomally-located *bont* genes has yet to be definitively demonstrated.

It is thought that movement of *bont* gene clusters into non-neurotoxigenic clostridia is a one-way process that occurs via introduction within extrachromosomal plasmids followed by chromosomal integration. Previously published information and results from analysis of *bont* gene cluster locations in this study provide some evidence for this hypothesis. The ease of movement of plasmids containing *bont* genes among *C. parabotulinum* has been demonstrated in the laboratory and plasmid losses, with subsequent reversion to non-neurotoxicity, have also been noted. With the exception of a few very recently published genomes, there is no evidence of the persistence of these large plasmids after loss of their *bont* genes. While identical *bont* gene clusters have been located within either plasmids or the chromosome, the majority of *C. parabotulinum* strains (∼90%) contain chromosomally located *bont* gene clusters, indicating an ease of integration and/or selective pressure to do so. In strains where *bont* genes remain within extrachromosomal plasmids, targeting or facilitating genes that are necessary for chromosomal integration appear to be lacking. For example, strains having chromosomally located *bont/A2* gene clusters contain paired *arsC* genes (*arsC1* and *arsC2*) but in strains where *bont/A2* genes are extrachromosomal, *arsC1* genes are missing.

However, a question arises as to whether chromosomally integrated genes may be excised from the chromosome and re-inserted into the plasmid. In some cases, there is evidence of deterioration of the facilitating genes or IS elements that may have participated in chromosomal integration, so that use of these facilitators to reverse the integration process is not possible. In others, the plasmids have been lost. There are very few examples where *bont* gene clusters are located within the chromosome but an extracellular plasmid remains. In BoNT/A2F5 strains and the Australian BoNT/A2B6 strain AM526, *bont/F5* and *bont/B6* gene clusters remain within the plasmids but the *bont/A2* gene clusters are within the chromosome. In the former case, the *bont/F5* gene cluster inhabits the plasmid site where the *bont/A2* gene would be located, presumably blocking re-integration, but in the BoNT/A2B6 strain, the *HepA/SNF* site is present in the plasmid. It is not possible to determine if the chromosomally located *bont/A2* gene cluster in the BoNT/A2B6 strain could be re-integrated into the plasmid or not, as currently only one such strain has been isolated. The rarity of isolates where there is even a possibility for plasmid re-integration suggests chromosomal integration of *bont* gene clusters is essentially a one-way process, but further investigations are needed to confirm or refute this hypothesis.

While the genomic diversity seen within this species may have evolved partly through individual genetic mutations that over time have resulted in minor changes in genes, the major evolutionary driver behind genomic diversity is likely wholesale movements of individual genes, gene clusters, and large segments of DNA via horizontal gene transfers and recombination events. Analysis of recently sequenced *orfX C. parabotulinum* genomes from southern hemisphere bacterial collections have added to our overall knowledge about the BoNT-producing clostridia, increased our understanding of the diversity seen among these strains, and provided insights into mechanisms behind the generation of this diversity. Bacterial strains have been transported across oceans and continents using various environmental means and interactions between diverse strains have produced novel genomic variants. Over time, gene transfers and exchanges have shaped *bont* gene diversity. While these observations are focused on a particular subset of a single bacterial species, insights may be applied to the study of diversity within other clostridial species and possibly other bacteria as well.

## Data Availability Statement

NCBI accession information for all genomic data presented in this study is available in the Supplementary Material.

## Author Contributions

TS performed analyses and wrote the manuscript. CW performed analyses and edited the manuscript. JS edited the manuscript and guided genomic analyses. KH, SJ, FA, BA, RF, PC, and PK contributed strains/DNA/genomic sequencing and source information for strains. All authors reviewed the manuscript.

## Conflict of Interest

The authors declare that the research was conducted in the absence of any commercial or financial relationships that could be construed as a potential conflict of interest.

## Footnotes

1 https://www.selectagents.gov/selectagentsandtoxinslist.html

2 http://tree.bio.ed.ac.uk/software/figtree/
